# A Sialoreceptor Binding Motif in the *Mycoplasma synoviae* Adhesin VlhA

**DOI:** 10.1371/journal.pone.0110360

**Published:** 2014-10-22

**Authors:** Meghan May, Dylan W. Dunne, Daniel R. Brown

**Affiliations:** 1 Department of Biomedical Sciences, College of Osteopathic Medicine, University of New England, Biddeford, Maine, United States of America; 2 Department of Biological Sciences, Jess and Mildred Fisher College of Science and Mathematics, Towson University, Towson, Maryland, United States of America; 3 Department of Infectious Diseases and Pathology, College of Veterinary Medicine, University of Florida, Gainesville, Florida, United States of America; Miami University, United States of America

## Abstract

*Mycoplasma synoviae* depends on its adhesin VlhA to mediate cytadherence to sialylated host cell receptors. Allelic variants of VlhA arise through recombination between an assemblage of promoterless *vlhA* pseudogenes and a single transcription promoter site, creating lineages of *M. synoviae* that each express a different *vlhA* allele. The predicted full-length VlhA sequences adjacent to the promoter of nine lineages of *M. synoviae* varying in avidity of cytadherence were aligned with that of the reference strain MS53 and with a 60-a.a. hemagglutinating VlhA C-terminal fragment from a Tunisian lineage of strain WVU1853^T^. Seven different sequence variants of an imperfectly conserved, single-copy, 12-a.a. candidate cytadherence motif were evident amid the flanking variable residues of the 11 total sequences examined. The motif was predicted to adopt a short hairpin structure in a low-complexity region near the C-terminus of VlhA. Biotinylated synthetic oligopeptides representing four selected variants of the 12-a.a. motif, with the whole synthesized 60-a.a. fragment as a positive control, differed (*P*<0.01) in the extent they bound to chicken erythrocyte membranes. All bound to a greater extent (*P*<0.01) than scrambled or irrelevant VlhA domain negative control peptides did. Experimentally introduced branched-chain amino acid (BCAA) substitutions Val3Ile and Leu7Ile did not significantly alter binding, whereas fold-destabilizing substitutions Thr4Gly and Ala9Gly tended to reduce it (*P*<0.05). Binding was also reduced to background levels (*P*<0.01) when the peptides were exposed to desialylated membranes, or were pre-saturated with free sialic acid before exposure to untreated membranes. From this evidence we conclude that the motif P-X-(BCAA)-X-F-X-(BCAA)-X-A-K-X-G binds sialic acid and likely mediates VlhA-dependent *M. synoviae* attachment to host cells. This conserved mechanism retains the potential for fine-scale rheostasis in binding avidity, which could be a general characteristic of pathogens that depend on analogous systems of antigenically variable adhesins. The motif may be useful to identify previously unrecognized adhesins.

## Introduction

The bacterial pathogen *Mycoplasma synoviae* is associated with a broad spectrum of clinical manifestations ranging from inapparent infection to systemic disease of poultry. Infection is most commonly associated with inflammatory lesions of the joints, respiratory and/or reproductive tract and results in reduced feed conversion and poor egg quality. Less commonly, *M. synoviae* can be found infecting additional tissues in galliform birds (*e.g.* spleen, liver, central nervous system, skeletal muscle, and eye) [1]–[4] and respiratory tissues or synovial membranes of distantly related avian species such as ducks, geese, pigeons, and sparrows [5].

Attachment to sialylated receptors on host cells is mediated by the *M. synoviae* variable lipoprotein hemagglutinin VlhA [6]–[7]. Previous analyses indicated that the *vlhA* gene family has been laterally transferred between *M. synoviae* and *Mycoplasma gallisepticum* possibly during coinfection of a shared avian host [8]–[9]. In *M. synoviae*, antigenic variants of this adhesin result from unidirectional recombination between a single expression site and a large reservoir of *vlhA* pseudogenes [10]. In contrast, altered expression in *M. gallisepticum* stems from the expansion and contraction of a poly-GAA repeat upstream of the promoters of each copy of *vlhA* [11]. The selective pressure of specific host immune responses to these antigens is thought to drive diversity in *vlhA* allele expression [10]–[13]. Despite the critical importance of cytadherence to the establishment and maintenance of infection, discrete VlhA types were demonstrated to have significantly different avidities for host cell binding, which can be quantified by agglutination of erythrocytes [14]. *M. synoviae*'s capacity for cytadherence maps surprisingly to a hypervariable C-terminal domain of VlhA called MSPA [15]–[16]. The precise means of attachment and how this capacity is retained despite such extensive sequence polymorphism and allele switching are not known. We sought to identify and characterize the specific motif that mediates adhesion of VlhA proteins to host cells.

## Materials and Methods

### Identification and Structural Modeling of the Putative Hemagglutination Motif (PHM)

The predicted full-length VlhA sequences adjacent to the single transcription promoter of nine lineages of *M. synoviae* varying in avidity of cytadherence (F10-2AS, FMT, K4907, K5016, K5395, MS117, MS173, MS178, and a>30X-passaged Florida lineage of strain WVU1853^T^) [14] were aligned with that of the reference strain MS53 [8] and with a 60-a.a. hemagglutinating VlhA C-terminal fragment from a ca. 12X-passaged Tunisian lineage of strain WVU1853^T^ [15] by using ClustalΩ [17]. The multiple alignment was manually inspected for conserved motifs, evident as contiguous residues inferred to be under stabilizing selection (ω<1) by using Bayesian models of sequence evolution in the Selecton v2.4 software suite [18]. The secondary structures of full-length VlhA, MSPA and its C-terminal 60 residues, and of the putative hemagglutination motifs (PHMs) described were modeled using the Phyre2 suite of template-directed and *ab initio* protein structure prediction algorithms (http://www.sbg.bio.ic.ac.uk/phyre2) [19]. The effects of individual amino acid substitutions on peptide structural stability were predicted by applying the Site Directed Mutator algorithm (http://mordred.bioc.cam.ac.uk/~sdm/sdm.php) [20] to the.pdb files generated by Phyre2. Substitutions having stability scores (ΔΔG) between −0.5 and 0.5 were predicted to be neutral, whereas those <−2 or>2 were predicted to be highly destabilizing. The potential to bind sialic acid (KEGG Compound C00270; PubChem.sdf 445063) or any other ligand in the KEGG Compound database was predicted by applying the eFindSite ligand binding site prediction algorithm (http://brylinski.cct.lsu.edu/) [21]–[22] also to the.pdb files generated by Phyre2.

### Quantitative Binding of PHM Peptides

**Figure 1:**
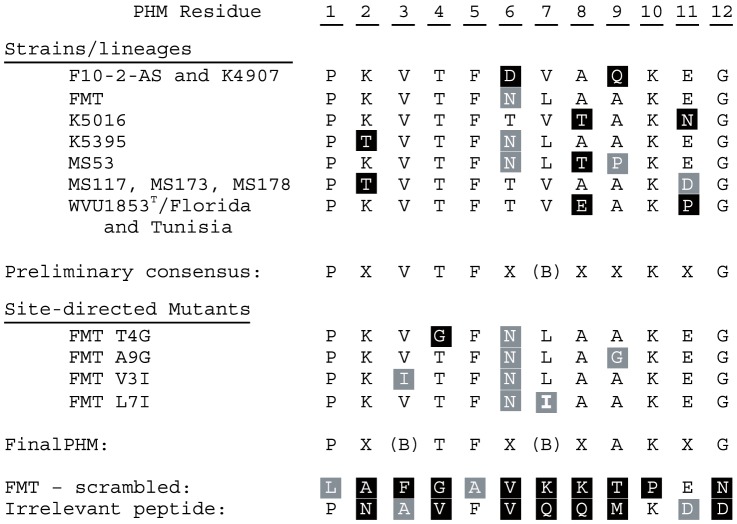
Aligned PHM and control peptide sequences. The putative hemagglutination motif (PHM) was deduced by aligning the adhesin protein VlhA allele present at the expression site of ten specimens of *M. synoviae* with a 60-a.a. hemagglutinating VlhA C-terminal fragment from the Tunisian lineage of strain WVU1853^T^, then inspecting the alignment for contiguous residues inferred to be under stabilizing selection. Peptides representing five variants of the PHM, including strains having a>20-fold range in quantitative hemagglutination phenotypes [14], were synthesized. Directed mutations were introduced at selected residues relative to the PHM from strain FMT, which had only one difference (Thr6Asn) from the most common amino acid at each residue. The mutations Val3Ile and Leu7Ile were predicted to be inconsequential, while Thr4Gly and Ala9Gly were predicted to affect PHM structure and/or function. Negative control peptides used in erythrocyte membrane-binding assays are also shown. Functionally non-synonymous differences relative to the most common amino acid at each residue are shaded in black, synonymous differences are shaded in gray, and identical residues are not shaded. (B)  =  branched chain amino acid.

Twelve-a.a. peptides representing five variants of the PHM from strains FMT, K5016, K5395, MS53 and WVU1853^T^, plus the whole 60-a.a. hemagglutinating fragment of the Tunisian lineage of strain WVU1853^T^, were synthesized, biotinylated and lyophilized (Biomatik, Wilmington, DE). Purity of each lyophilized preparation was confirmed by HPLC to be 90–92% full-length peptide. Those strains were chosen because FMT, K5016, K5395 and the Florida lineage of WVU1853^T^ spanned a>20-fold range in quantitative hemagglutination phenotypes, and the entire *vlhA* locus sequence of the reference strain MS53 has been published. [8], [14]. Peptides having single directed mutations introduced at the conserved residues 3 or 4, or non-conserved residues 7 or 9, were also synthesized using the strain FMT motif PKVTFNLAAKEG as a parent. FMT was chosen as the parent motif because it had only one difference (Thr6Asn) from the most commonly observed amino acid at each residue (Figure 1). The functionally synonymous substitutions Val3Ile and Leu7Ile (BLOSUM62 [23] scores>0) were predicted to be inconsequential, while non-synonymous Thr4Gly and Ala9Gly (BLOSUM62 scores ≤0) were predicted to affect PHM structure and/or function.

The capacity of the peptides to bind to native or desialylated chicken erythrocyte membranes was assessed quantitatively in an ELISA format. Microtiter plates were coated with 5% v/v suspensions of chicken erythrocytes (Lampire Biologicals, Pipersville, PA) diluted 1∶3 in 0.5 M sodium bicarbonate lysis buffer, pH 10.0, to a total volume of 300 µL per well. Desialylated membranes were prepared by pre-treatment of the erythrocytes with 10 U/ml of sialidase purified from *Clostridium perfringens* (Sigma-Aldrich, St. Louis, MO) for 1 hr at 37°C. Following coating for 12 hr at 4°C, cellular debris including hemoglobin was removed by washing each well 3× with 300 µL of PBS, pH 7.4, and sealed plates were blocked 1 hr at 37°C with 300 µL per well of 5% v/v fetal bovine serum in PBS.

After washing the membrane-coated and blocked wells 3× with 300 µL of PBS, 50 µg of biotinylated peptide solubilized in 50 µL of water was added to each of duplicate wells and allowed to bind for 1 hr at 37°C. After washing each well 3× with 300 µL of PBS, bound peptides were detected using horseradish peroxidase-conjugated streptavidin (2 µg/mL, Sigma-Aldrich, St. Louis, MO) and the chromogenic substrate 3,3′,5,5′-tetramethylbenzidine (Thermo Fisher Scientific, Waltham, MA) with an acid stop followed by spectrophotometric analysis (λ = 450 nm). The hemagglutinating 60-mer of the Tunisian lineage of strain WVU1853^T^ served as the positive control peptide, and negative controls were a scrambled version of the PHM from strain FMT (LAFGAVKKTPEN) and an irrelevant peptide (PNAVFVQQMKDD) from a distant site in the expressed VlhA of the Florida lineage of strain WVU1853^T^ (GenBank AEA01932.1). The effect of pre-saturation with ligand was tested by first incubating the peptides in 250 mg/ml N-acetylneuraminic acid (Sigma-Aldrich, St. Louis, MO) in water without pH adjustment at a peptide: ligand molar ratio of 1∶2×10^4^ for 1 hr at 37°C.

### Statistical Procedures

The effect of peptide sequence on extent of adherence to membranes (*n* = 3 independent replications of each treatment combination, with duplicate measurements of each peptide within replicate) was analyzed by ANOVA, with Tukey-Kramer Honestly Significant Difference (HSD) post-hoc comparisons used to group the means when the main effect was significant (*P*<0.05 or less). The effects of membrane pre-treatment with sialidase and peptide pre-saturation with sialic acid were analyzed by ANOVA, with HSD or Dunnett's post-hoc comparisons to the corresponding native specimens when the main effect was significant. Statistical analyses were performed using Origin 9 (OriginLab, Northampton, MA) software.

### Motif Distribution in *M. synoviae* and *M. gallisepticum*

*M. synoviae* strain MS53 *vlhA* pseudogene sequences and *M. gallisepticum* strains R, F, WI01, NY01, NC06, CA06, VA94, NC95, NC08, and NC96 were obtained from GenBank (accession numbers NC_007294.1, NC_004829.2, NC_017503.1, NC_018410.1, NC_018409.1, NC_018411.1, NC_018412.1, NC_018406.1, NC_018407.1, NC_018413.1, and NC_018408.1, respectively). Occurrences of PHM-encoding sequences were totaled and normalized to the total length of *vlhA*-encoding sequence in each strain. Each member of the *vlhA* pseudogene reservoir of *M. synoviae* strain MS53 was used to construct a neighbor-joining tree (bootstrap n = 100) using ClustalW2 [24]. The designated outgroup was *vlhA* 4.02 from *M. gallisepticum*.

## Results

### Identification of the PHM

**Figure 2:**
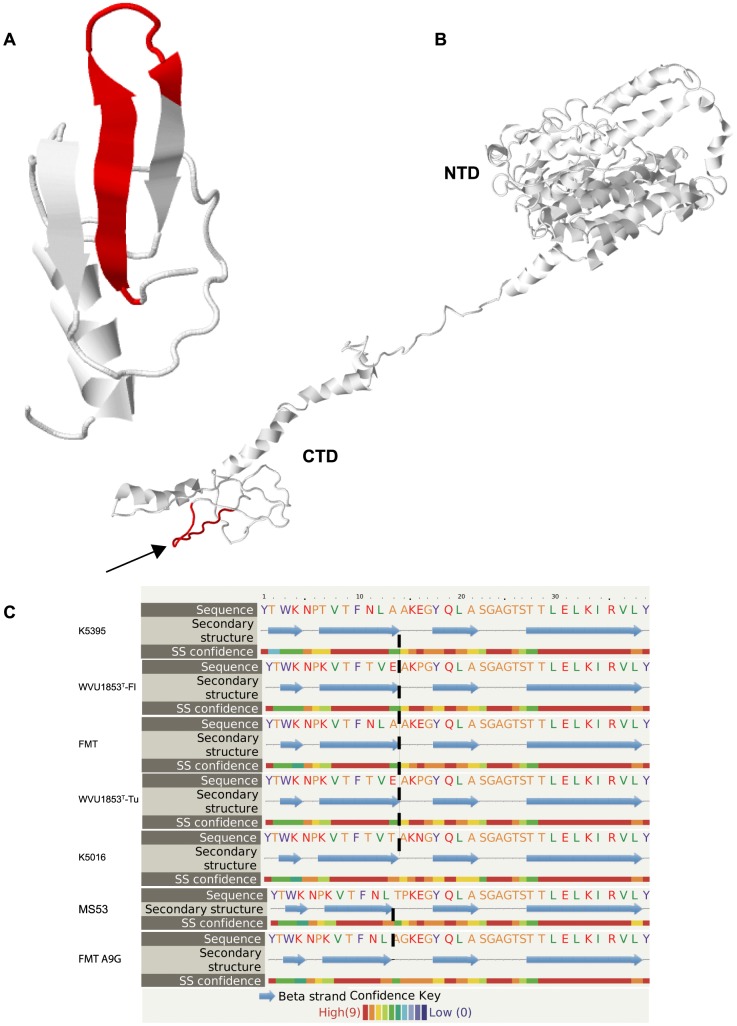
PHM structural predictions. (**A**) The putative hemagglutination motif (PHM; red) was predicted to adopt a hairpin structure of two anti-parallel β strands separated by a short disordered loop. (**B**) The motif (red, indicated by arrow) mapped to a low-complexity region near the carboxyterminal domain (CTD) of the *M. synoviae* adhesin protein VlhA cleavage product MSPA, shown here in the structure predicted for the Tunisian lineage of strain WVU1853^T^. The N-terminal domain (NTD) of MSPA was predicted to have much greater 3-dimensional complexity. (**C**) The length of the disordered loop was predicted to be longer in PHM peptides that bound to avian erythrocyte membranes (representing Florida and Tunisian lineages of strain WVU1853^T^ and strains FMT, K5016 and K5395) than in the reduced-binding peptide mutant FMT-Ala9Gly and the non-binding peptide representing strain MS53.

When the full-length expressed VlhA protein MSPA sequences of nine strains of *M. synoviae* that vary in avidity of cytadherence were aligned with MSPA of the reference strain MS53 [8] and a 60-a.a. hemagglutinating peptide derived from the C-terminus of MSPA expressed by the Tunisian lineage of strain WVU1853^T^ [15], an imperfectly conserved 12-a.a. motif was evident in all sequences (Figure 1). A total of seven different PHM sequence variants were evident among the 11 total sequences aligned. Strains FMT, K5016, K5395 and MS53 all had unique PHM sequences; the sequences in strains F10-2-AS and K4907 were identical; the sequences in Florida and Tunisian lineages of WVU1853^T^ were identical; and the sequences in Argentine strains MS117, MS173 and MS178 were all identical. Six of twelve residues in the PHM were perfectly conserved across strains, two (residues 6 and 7) were conserved in polarity and hydrophobicity, respectively, and four were variable. Polar Asn_6_ or Asp_6_ were invariably paired with Leu_7_, while Thr_6_ was invariably paired with Val_7_. The motif was predicted to adopt a short hairpin secondary structure of two anti-parallel beta strands, separated by a disordered loop of four or five residues, in a region of low structural complexity (regional structure prediction confidence <70%) near the C-terminus of MSPA (Figure 2a, b). Fifty-three percent of residues in the full-length VlhA were modeled at>90% confidence [19], with the regions of greatest confidence being similar to the streptococcal adhesin emb (99.8% confidence) and the staphylococcal extracellular matrix-binding protein ebhA (99.4%). The degree of structural complexity in the C-terminus of MSPA was otherwise too low for the algorithms to predict binding of any specific ligand.

**Figure 3:**
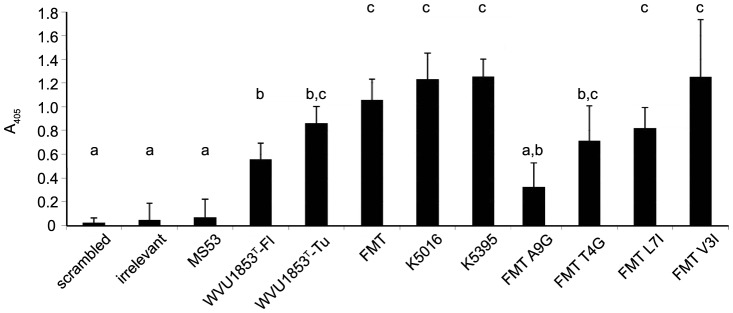
Erythrocyte membrane binding by PHM peptides. Bars depict mean ± standard error of the amount of synthetic peptide bound to avian erythrocyte membranes in an ELISA format (n = 3 independent replicates, with duplicate measurements of each peptide within replicate). The peptides represented variants of the putative hemagglutination motif (PHM) at the VlhA expression site of *M. synoviae* strains MS53, WVU1853^T^ (Florida and Tunisian lineages), FMT, K5016 and K5395, which spanned a>20-fold range in quantitative hemagglutination phenotypes [14]. The positive control was the Tunisian lineage of strain WVU1853^T^, and negative controls were scrambled strain FMT peptide and an irrelevant peptide from a distant site in VlhA from the Florida lineage of strain WVU1853^T^. Different letters above the bar indicate means that differ (*P*<0.05 or less) by Tukey-Kramer Honestly Significant Difference test. As predicted, the directed substitution Ala9Gly significantly reduced binding versus the parent peptide from strain FMT, and Thr4Gly tended to reduce binding, whereas Val3Ile and Leu7Ile did not significantly alter binding.

Synthetic biotinylated peptides representing the full-length 60-a.a. hemagglutinating fragment and four strain variants of its candidate 12-a.a. cytadherence motif (Figure 1) bound to chicken erythrocyte membranes in an ELISA format and could be detected by probing with horseradish peroxidase-conjugated streptavidin. Four of the peptides bound to membranes to a significantly greater extent (*P*<0.05) than scrambled or irrelevant control peptides did, but a peptide representing the corresponding motif from strain MS53 did not bind to membranes to any extent greater than background (Figure 3). Single neutral substitutions (predicted ΔΔG  = −0.25) experimentally introduced at conserved residue 3 (Val3Ile) or non-conserved residue 7 (Leu7Ile) did not alter binding to membranes with respect to the extent of binding by the parent motif of strain FMT, whereas the experimental destabilizing substitution Thr4Gly (predicted ΔΔG  = −2.31) tended to reduce binding (Figure 3). The motif of strain MS53 differs naturally from all others by Ala9Pro (BLOSUM62  = −1; predicted ΔΔG  = −2.22), and the even more destabilizing substitution Ala9Gly (predicted ΔΔG  = −3.88) nearly abolished binding when introduced into the parent motif of strain FMT (*P*<0.05; Figure 3). These effects correlated with a predicted change in length of the disordered loop in the hairpin secondary structure of the motif (Figure 2c).

**Figure 4:**
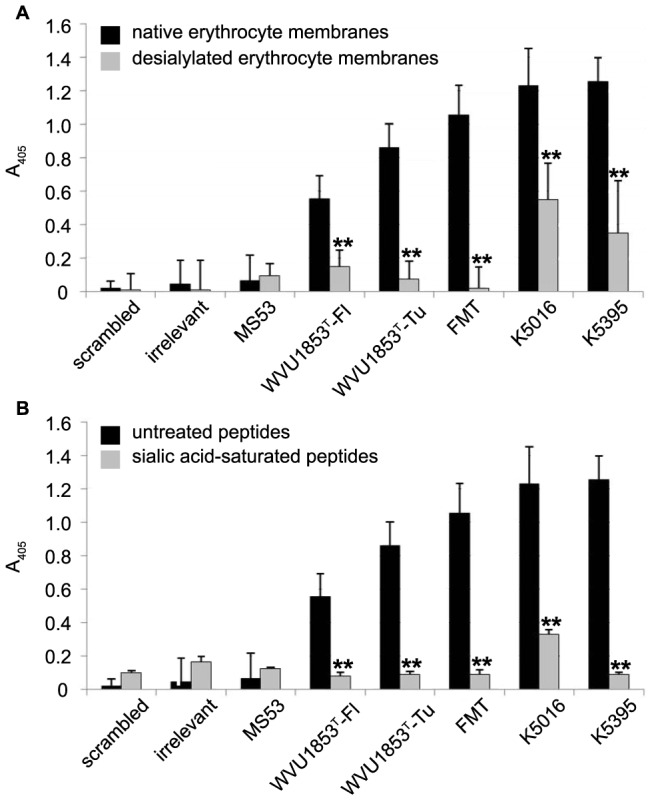
Effects of sialylation and desialylation on PHM peptide binding. Bars depict mean ± standard error of the amount of synthetic peptide bound to avian erythrocyte membranes in an ELISA format (n = 3 independent replicates, with duplicate measurements of each peptide within replicate). (**A**) Desialylation of erythrocyte membranes significantly reduced PHM peptide binding relative to native membranes (**  = *P*<0.01) for all strains of *M. synoviae* except MS53, which bound to native or desialylated erythrocyte membranes at background levels. (**B**) Presaturation of PHM peptides with free sialic acid before exposure to native erythrocyte membranes significantly reduced binding relative to untreated peptides (**  = *P*<0.01) for all strains except MS53, on which sialic acid had no effect.

Binding of the peptides to desialylated membranes was significantly reduced (*P*<0.01) relative to untreated membranes for all peptides except those representing strain MS53 and the scrambled and irrelevant controls (Figure 4a). When pre-incubated with free sialic acid, all peptides except the one representing strain MS53 and the scrambled and irrelevant controls had significantly diminished (*P*<0.01) capacity for membrane binding (Figure 4b). From this evidence we conclude that the composite amino acid motif P-X-(BCAA)-X-F-X-(BCAA)-X-A-K-X-G binds sialic acid and likely mediates VlhA-dependent *M. synoviae* attachment to sialylated receptors on the surface of avian erythrocytes.

### PHM Distribution among *M. synoviae* Strain MS53 *vlhA* Pseudogenes and *Mycoplasma gallisepticum vlhA* Homologs

**Figure 5:**
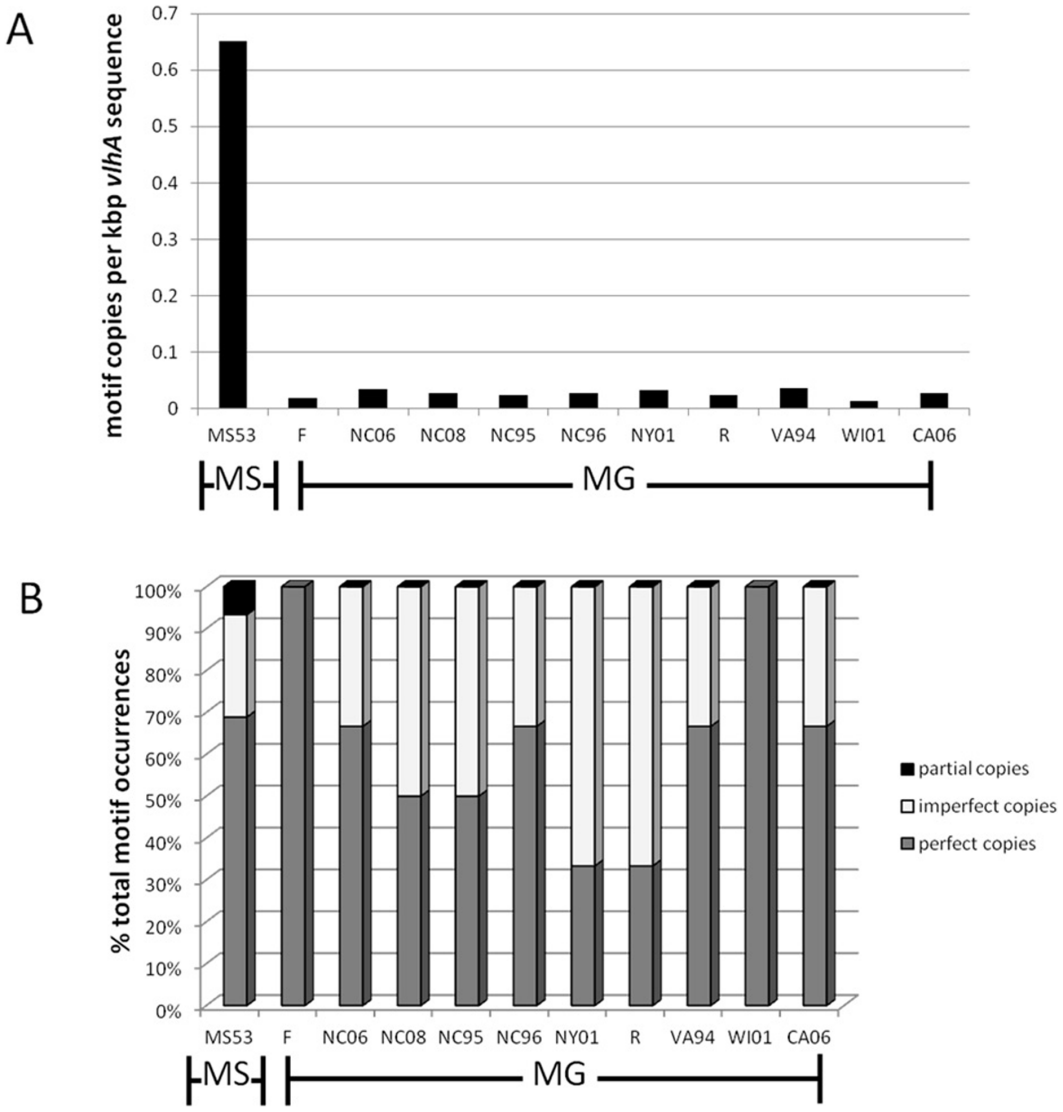
PHM distribution in *vlhA* genes and pseudogenes in *M. synoviae* and *M. gallisepticum*. (**A**) PHM-encoding sequence as a function of total kbp of *vlhA* sequence is elevated 18-fold in *M. synoviae* (MS) reference strain MS53, the only strain for which the entire *vlhA* locus sequence has been published, relative to 10 fully-sequenced strains of *M. gallisepticum* (MG), evidence that it is far more common in *M. synoviae*. (**B**) The relative proportions of perfect and imperfect PHM copies were comparable between strains of *M. synoviae* and *M. gallisepticum*.

Candidate PHM sequences occurred in 45 of the 70 putative *vlhA* pseudogenes of *M. synoviae* strain MS53 [8], 39% of the time with no deviation from the consensus among the alleles expressed by the strains examined, 20% with a single deviation, and 17% with two deviations from consensus. Phylogenetic clustering of *vlhA* pseudogenes containing intact copies of the PHM did not correlate with their syntenic order in the strain MS53 genome (Figure S1). The PHM occurred at least 18-fold more frequently in strain MS53 (0.65 motifs/kb of *vlhA* sequence) than in the genomes of any of 10 strains of *M. gallisepticum* (0.014–0.037 motifs/kb of *vlhA* sequence), a species known to employ a different primary cytadherence mechanism [25] (Figure 5a). The rate of occurrence of imperfect PHMs was comparable between the two species (Figure 5b).

## Discussion

One of the defining moments of many infections is the attachment of a disease-causing agent to its host. Understanding how the parasitic bacterial species *M. synoviae* colonizes a host cell's surface is paramount to understanding how to prevent infection. It is known that the protein family VlhA is responsible for attachment by *M. synoviae*, but the functional motifs of the adhesin and the molecular basis for rheostasis in binding avidity have not been characterized. Proteins in this family from multiple strains of *M. synoviae* have been identified as having a role in the attachment to host blood cells [7], [15]. Khiari *et al*. [15] mapped the capacity for attachment to the carboxyterminus of VlhA, and we utilized that finding to identify a specific motif sufficient to mediate VlhA binding to sialylated host cells.

Sequence conservation across adherent strains enabled the identification of a 12-residue putative hemagglutination motif that could be characterized further. This motif was predicted to have remarkably little structural complexity, in contrast to the complex topology of sialic acid ligand-binding domains of other microbes [26]–[27]. While residues at PHM positions 3 and 7 were conserved, substitution with similar residues having BLOSUM62 scores>0 did not alter function. The conserved Thr residue at position 4 could be changed to the dissimilar residue Gly (BLOSUM62  = −3) without loss of function. It is thus likely that the binding mechanism will tolerate synonymous substitutions at positions 3 and 7, and nonsynonymous substitutions at position 4. Residue 9 was a conserved Ala in all adherent strains. Strain MS53, which has an unknown attachment phenotype but is an attenuated strain, had the nonsynonmymous substitution Ala9Pro (BLOSUM62  = −1). Changing the strain FMT peptide to Gly_9_ (BLOSUM62  = 0) significantly diminished binding, and the strain MS53 peptide was non-adherent. Taken together, these results indicate that Ala_9_ is critical to PHM domain function. Our results indicate that the composite amino acid motif P-X-(BCAA)-X-F-X-(BCAA)-X-A-K-X-G mediates MSPA binding to avian erythrocytes. The potential to accommodate all amino acids with BLOSUM62 scores>0 at PHM positions 3 and 7 (*i.e.*, Ala, Met, Thr and Met, Phe, respectively) rather than restricting the parameters to branched-chain amino acids (Ile, Leu, Val) merits further analysis.

Previous studies indicated that whole *M. synoviae* cells interact with sialylated host cell receptors in order to facilitate attachment. Extrapolation from PHM peptide-binding to whole cell attachment necessarily requires demonstration of peptide-sialic acid interactions. Desialylation of avian erythrocytes prior to antigen preparation resulted in significant losses of binding capacity for all PHM peptides except the scrambled and irrelevant controls and strain MS53, for which desialylation had no effect on binding. In a reciprocal experiment, pre-adsorption of peptides with free sialic acid prior to exposure to intact erythrocyte antigen similarly diminished binding capacity for all PHM peptides except the scrambled and irrelevant controls and strain MS53. These results indicate a specific interaction between sialic acid and the PHM and support the hypothesis that the PHM domain mediates attachment of whole *M. synoviae* cells to host sialoreceptors.

The occurrance of PHM domains was not uniform among the pseudogenes of *M. synoviae* strain MS53, the only strain for which the entire pseudogene reservoir has been sequenced [8]. A majority (69%) of pseudogenes had perfect or near-perfect PHMs, while 31% had no discernible PHMs. To provide some context for the distribution of PHM domains in the sample of VlhA sequences existing within *M. synoviae* strain MS53, we examined the frequency and distribution in an alternative sample of VlhA sequences that exist distributed across multiple strains of *M. gallisepticum*. In contrast to the 45 copies in *M. synoviae* strain MS53, sequenced *M. gallisepticum* strains ranged from having just a single copy of *vlhA* encoding a PHM domain (strains WI01 and F) up to a maximum of only 3 copies (strains R, NY01, NC06, CA06, VA94, NC95, and NC96). Normalization to the total amount of *vlhA* sequence within species confirmed that *M. synoviae* has a greatly elevated instance of PHM-encoding sequence relative to *M. gallisepticum*, and that the low frequency of PHM is consistent across strains of *M. gallisepticum*. The multiple independent cytadherence mechanisms of *M. gallisepticum* [28]–[32] may allow the decay of PHM domains within VlhA proteins, while selective pressure to retain the functional motif in the homologous proteins in *M. synoviae* is substantially greater due to the absence of other mechanisms of cytadherence.

This work describes a novel functional motif associated with adherence to sialic acid, and its distribution across *vlhA* pseudogenes. This very specific protein fragment pattern may be a target to design novel drug therapies or vaccines to alleviate or prevent infection due to *M. synoviae* as well as other pathogens that use similar mechanisms to attach to their hosts, and allows for the identification of currently unrecognized microbial adhesins targeting sialoreceptors.

## Supporting Information

Figure S1 **Distribution and relatedness of PHM-encoding pseudogenes.** PHM-encoding pseudogenes (shaded) did not cluster together as a separate group from non-encoding pseudogenes. Relatedness of pseudogenes did not reflect gene synteny.
(TIF)
